# Diagnostic and prognostic significance of circulating secreted frizzled‐related protein 5 in colorectal cancer

**DOI:** 10.1002/cam4.7352

**Published:** 2024-06-14

**Authors:** Runhao Li, Saifei Liu, Kenny Yeo, Suzanne Edwards, Man Ying Li, Ryan Santos, Sima Kianpour Rad, Fangmeinuo Wu, Guy Maddern, Joanne Young, Yoko Tomita, Amanda Townsend, Kevin Fenix, Ehud Hauben, Timothy Price, Eric Smith

**Affiliations:** ^1^ Solid Tumour Group, Basil Hetzel Institute for Translational Health Research The Queen Elizabeth Hospital Woodville South South Australia Australia; ^2^ Adelaide Medical School The University of Adelaide Adelaide South Australia Australia; ^3^ Discipline of Surgery, The University of Adelaide, Basil Hetzel Institute for Translational Health Research, The Queen Elizabeth Hospital Woodville South South Australia Australia; ^4^ School of Public Health The University of Adelaide Adelaide South Australia Australia; ^5^ Viral Immunology Group The University of Adelaide and Basil Hetzel Institute for Translational Health Research Woodville South Australia Australia; ^6^ Medical Oncology, The Queen Elizabeth Hospital Woodville South Australia Australia

**Keywords:** colorectal cancer, diagnosis, plasma biomarker, prognosis, SFRP5

## Abstract

**Background:**

Secreted Frizzled‐Related Protein 5 (SFRP5) modulates Wnt signalling pathways, affecting diverse biological processes. We assessed the diagnostic and prognostic value of circulating SFRP5 (cSFRP5) in colorectal cancer (CRC)

**Methods:**

Plasma cSFRP5 concentrations were measured using enzyme‐linked immunosorbent assay (ELISA) in healthy donors (*n* = 133), individuals diagnosed with CRC (*n* = 449), colorectal polyps (*n* = 85), and medical conditions in other organs including cancer, inflammation, and benign states (*n* = 64).

**Results:**

Patients with CRC, polyps, and other conditions showed higher cSFRP5 levels than healthy individuals (*p* < 0.0001). Receiver operating characteristic curves comparing healthy donors with medical conditions, polyps and CRC were 0.814 (*p* < 0.0001), 0.763 (*p* < 0.0001) and 0.762 (*p* < 0.0001), respectively. In CRC, cSFRP5 correlated with patient age (*p* < 0.0001), tumour stage (*p* < 0.0001), and histological differentiation (*p* = 0.0273). Levels, adjusted for patient age, sex, plasma age and collection institution, peaked in stage II versus I (*p* < 0.0001), III (*p* = 0.0002) and IV (*p* < 0.0001), were lowest in stage I versus III (*p* = 0.0002) and IV (*p* = 0.0413), with no difference between stage III and IV. Elevated cSFRP5 levels predicted longer overall survival in stages II‐III CRC (univariate: HR 1.82, 95% CI: 1.02–3.26, *p* = 0.024; multivariable: HR 2.34, 95% CI: 1.12–4.88, *p* = 0.015).

**Conclusion:**

This study confirms cSFRP5 levels are elevated in CRC compared to healthy control and reveals a correlation between elevated cSFRP5 and overall survival in stages II‐III disease.

## 1 INTRODUCTION

Colorectal cancer (CRC) represents a significant global healthcare challenge, ranking as the third most diagnosed cancer and a leading cause of cancer‐related mortality.^1^ With over 1.8 million reported cases and more than 0.9 million deaths in 2020 alone, there is urgency for the development of early detection strategies and to significantly improve treatment efficacy and survival rates. Enhancing screening methodologies, advocating regular screenings among high‐risk populations, and developing precise, non‐invasive screening technologies are imperative.^2^, ^3^

Secreted frizzled‐related protein 5 (SFRP5) belongs to the SFRP family, an evolutionarily conserved group of extracellular glycoproteins crucial for modulating Wnt signalling pathways.^4^ These pathways play pivotal roles in various cellular processes, including embryonic development,^5^ tissue homeostasis, and the modulation of tumour initiation, growth, and metastasis.^6^, ^7^ Structurally, SFRPs contain an amino‐terminal signal peptide, a cysteine‐rich domain (CRD), and a carboxy‐terminal netrin‐like domain (NTR).^8^ The CRD is similar to the Wnt ligand binding site found in Frizzled family of cell surface receptor proteins.^9^ The NTR domain is involved in protein–protein interactions and is thought to contribute to the function of SFRPs.^10^ This structure enables SFRPs to act as soluble antagonists of Wnt signalling by interacting with Wnt ligands and inhibiting activation of the Frizzled receptors (reviewed in^11^).

SFRP5, specifically, has been shown inhibit multiple Wnt ligands such as Wnt5A and Wnt11, thereby regulating both canonical and non‐canonical Wnt pathways.^12^, ^13^ Experimental evidence from murine disease models and studies on SFRP5 knockout mice suggests that secretion of SFRP5 by adipocytes prevents hepatic steatosis and metabolic dysfunction associated with liver fibrosis by modulating inflammatory cells within adipose tissue.^12^, ^14^ In humans, downregulation of SFRP5 contributes to pro‐inflammatory conditions in visceral adipose tissue, potentially exacerbating obesity‐related comorbidities.^15^

Indeed, circulating SFRP5 (cSFRP5) has emerged as a potential biomarker in various diseases, participating in diverse pathological processes.^16^, ^17^, ^18^ Typically, individuals with metabolic disorders like obesity, insulin resistance, and diabetes exhibit reduced levels of cSFRP5 compared to healthy controls.^16^, ^17^, ^18^, ^19^ Meanwhile, interventions leading to significant weight loss in overweight individuals are associated with increased cSFRP5 levels, further highlighting its role in metabolic regulation.^20^, ^21^, ^22^ These findings collectively suggest that higher cSFRP5 levels are associated with reduced risk of metabolic disorders in humans. Interestingly, metabolic syndrome has been shown to be associated with increased risk of liver metastasis in CRC patients.^23^ Additionally, cSFRP5 has implications in cardiovascular diseases, with lower concentrations observed in individuals with arterial stiffness,^24^ atherosclerosis,^25^ coronary artery disease^26^, ^27^ and hypertension.^21^, ^28^

In contrast, the role of cSFRP5 in cancer remains understudied. Our previous study indicated that cSFRP5 was elevated in CRC compared to non‐CRC controls, and elevated cSFRP5 levels were associated with extended disease‐free survival among patients in stages I‐III.^29^ Yet, limitations, such as small sample size and insufficient control groups, hindered the comprehensive understanding of its potential. In this study, we measured cSFRP5 concentrations in an independent, retrospective, multi‐centre cohort encompassing 133 healthy controls without documented pathology, 449 patients with different stages of CRC, 85 individuals with colorectal polyps, and 64 individuals with other notable medical conditions in other organs including cancer, inflammation, and benign states. Our primary objectives were to validate cSFRP5's diagnostic and prognostic potential and explore its correlation with CRC progression‐related clinical‐pathological parameters and overall survival. Our secondary objectives were to determine if cSFRP5 was altered in patients diagnosed with colorectal polyps and other medical conditions in other organs including cancer, inflammation and benign states.

## 2 MATERIALS AND METHODS

### 2.1 Patient plasma samples

All plasma samples were obtained from Victorian Cancer Biobank (Table 1), sourced from multiple institutions (Table S1) and collected using a standardised protocol to minimise variation between institutions. The age of the plasma samples was matched between the donor groups, with all samples collected over a period from 1999 until 2022, with the 133 healthy donors from 2004 to 2021, the 449 CRC from 1999 to 2021, the 85 polyps from 2003 to 2022, and the 64 with other notable medical conditions from 2002 to 2021. All CRC samples were collected pre‐operatively, prior to planned surgery.

**TABLE 1:** Summary of participants in this study.

Group	*n*	Female (%)	Median age, years (range)
Healthy (H)	133	86 (64.7%)	42 (16–80)
Notable medical conditions (D)	64	15 (23.4%)	60 (25–88)
Colorectal polyps (P)	85	40 (47.1%)	66 (22–88)
Colorectal cancer (CRC)			
Stage I	147	60 (40.8%)	68 (34–91)
Stage II	103	40 (38.8%)	68 (32–95)
Stage III	109	36 (33.0%)	68 (28–89)
Stage IV	90	36 (40.0%)	57.5 (25–82)
CRC total	449	172 (38.3%)	66 (25–95)
**Total**	**731**	**313 (42.8%)**	**63 (16–95)**

Bold values indicates statisically significant *p*‐values.

### 2.2 SFRP5 enzyme‐linked immunosorbent assay

The plasma cSFRP5 concentration was determined using a commercially sourced, commonly used enzyme‐linked immunosorbent assay (ELISA) according to the manufacturer's instructions (Cloud Clone Corp., TX, USA).^29^ All ELISA kits used in this study were sourced from a single lot number (Lot# L210917454), with a detection range of 1.56–100 ng/mL. Plasma samples were diluted 1 in 2 in phosphate buffered saline (pH 7.0–7.2) and analysed in duplicate. The optical density at 450 nm was measured using iMark Microplate Absorbance Reader (Bio‐Rad Laboratories, Inc., CA, USA), and the calculated cSFRP5 concentration was derived from the standard curve.

### 2.3 Statistical analysis

Statistical analyses were conducted using either Prism 10 for MacOS (GraphPad Software, Inc., CA, USA), SAS OnDemand for Academics (SAS Institute Inc., NC, USA), or the R package, survival v3.5. To assess differences in cSFRP5 concentration between patient groups, we utilised Kruskal–Wallis with Dunn's multiple comparisons test. Correlations between cSFRP5 concentration and various prognostic clinical‐pathological parameters were examined using the Mann–Whitney test. A linear regression model was employed to evaluate the relationship between cSFRP5 concentration and patient group, tumour invasion stage, TNM group, as well as other prognostic clinical‐pathological parameters. This model was adjusted for potential confounding variables, including patient age, sex, plasma age and collection institution. Assumptions crucial to the linear models, such as normality of residuals and homoscedasticity, were found to be upheld through careful inspection of residual histograms and scatterplots of residuals against predicted values. Univariate and multivariable Cox proportional hazard models were performed using R package, survival v3.5, to assess relationship between overall survival, cSFRP5 concentration, TNM stage, age, sex, vascular or perineural invasion, histological differentiation, and tumour site. A *p* ≤ 0.05 was deemed statistically significant.

## 3 RESULTS

### 3.1 Patient cohorts

The study included 731 participants (Table 1), comprising 133 healthy donors without documented pathology (Table S2), 449 individuals diagnosed with CRC, 85 individuals with colorectal polyps (Table S3), and 64 individuals with other notable medical conditions in other organs (Table S4). The polyps group comprised the following subtypes: villous/tubulovillus (*n* = 48), adenoma/adenomatous (*n* = 31), sessile serrated (*n* = 2), hyperplastic (*n* = 2), inflammatory (*n* = 1) and Peutz Jegher syndrome (*n* = 1). The other notable medical conditions included patients diagnosed with cancer (*n* = 4), chronic inflammation (*n* = 49), and benign diseases (*n* = 11). The healthy participants consisted of more females (*p* < 0.0001) and were significantly younger (*p* < 0.0001) (Figure S1). All participants attended one of eight healthcare institutions, and there were no discernible differences in cSFRP5 concentration observed among institutions within any of the donor groups (Figure S1). There were no correlations between the age of the plasma samples and cSFRP5 concentration in any of the donor groups (Table S5).

### 3.2 Circulating SFRP5 in colorectal cancer

**FIGURE 1:**
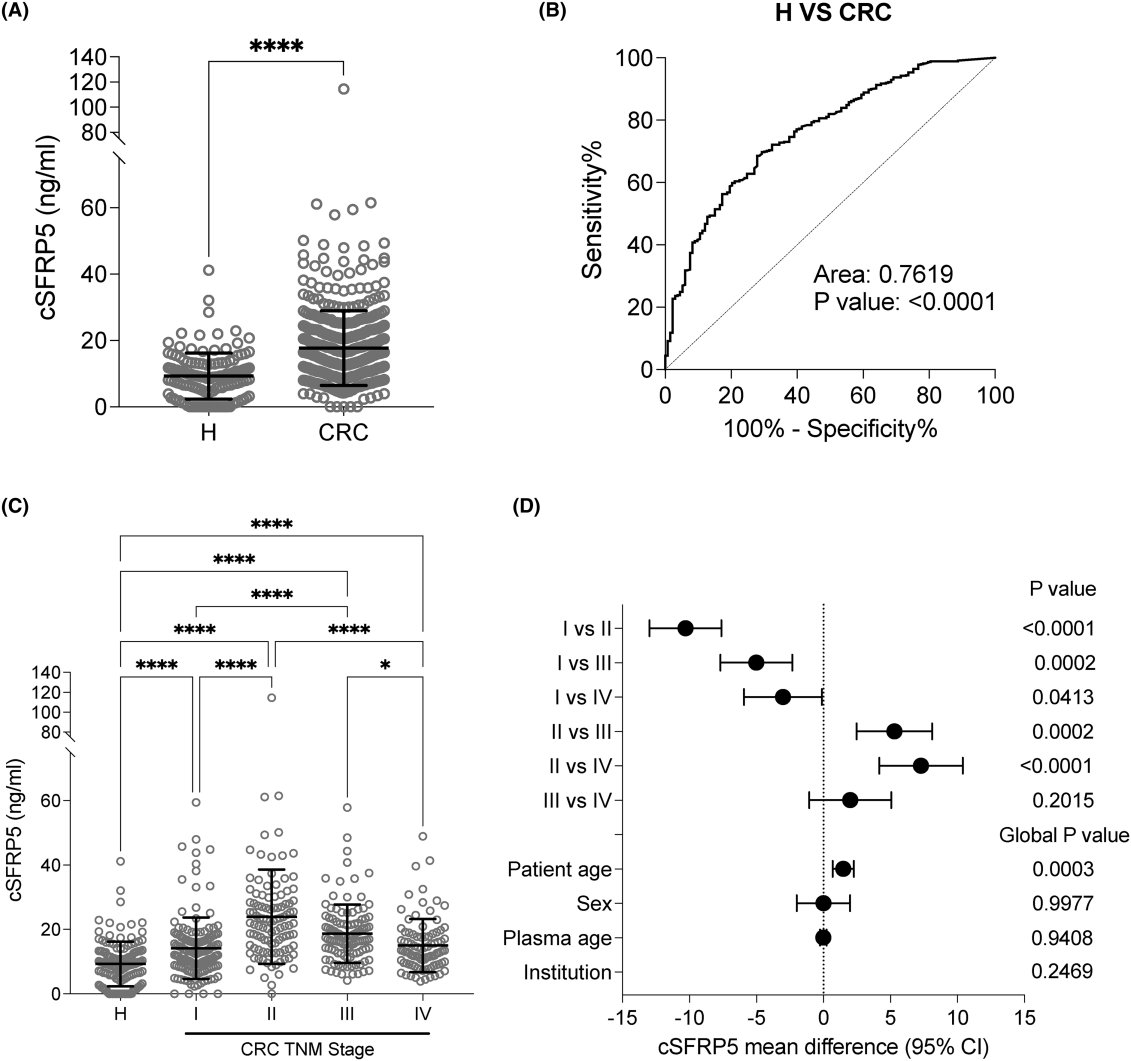
(A) Concentration of cSFRP5 in healthy donors (H) (*n* = 133) and patients with colorectal cancer (CRC) (*n* = 449). (B) AUROC curve for cSRFP5 concentration comparing H to CRC. (C) Concentration of cSFRP5 in healthy donors (H) (*n* = 133) and CRC patients by TNM stage I (*n* = 147), stage II (*n* = 103), stage III (*n* = 109), and stage IV (*n* = 90). The data are the mean ± SD. Kruskal–Wallis with Dunn's multiple comparisons were used, *p < 0.05, ****p < 0.0001. (D) Forest plots of the mean (95% CI) differences in cSFRP5 concentration between different TNM stages, as determined by a linear regression model adjusting for patient age, sex, plasma age and collection institution differences.

The cSFRP5 concentration was significantly higher in CRC (mean 17.67 ng/mL, 95% confidence interval (95% CI): 16.63–18.72 ng/mL) compared to healthy donors (9.26 ng/mL, 95% CI: 8.08‐10.45 ng/mL; *p* < 0.0001; Figure 1A). The area under the receiver operating characteristic (AUROC) curve for discriminating between CRC and healthy donors was 0.762 (95% CI: 0.717–0.807; *p* < 0.0001; Figure 1B). With regards to TNM stage, the cSFRP5 was significantly higher in all stages of CRC compared to healthy donors (Figure 1C). Notably, the cSFRP5 levels were highest in stage II compared to either stage I (*p* < 0.0001), III (*p* = 0.0003) or IV (*p* < 0.0001). The cSFRP5 was significantly elevated in stage III compared to stage I (*p* = 0.0007) and IV (*p* = 0.0054). There was no significant difference between stage I and IV. We next utilised a linear regression model to account for patient age, sex, plasma age, and institution differences (Figure 1D; Table S6). Our analysis revealed a correlation between age and increased cSFRP5 concentration, but no discernible disparity was observed between males and females, or with plasma age, or collection institution. Adjusting for these variables, we found that cSFRP5 levels peaked in stage II versus I (*p* < 0.0001), III (*p* = 0.0002) and IV (*p* < 0.0001), were lowest in stage I versus III (*p* = 0.0002) and IV (*p* = 0.0413), with no difference between stage III and IV.

### 3.3 Circulating SFRP5 in colorectal polyps

Elevation of cSFRP5 was also observed in colorectal polyps, with a mean concentration of 17.34 ng/mL (95% CI: 15.00–19.69 ng/mL) compared to healthy donors (Figure S2A; *p* < 0.0001). The AUROC curve for discriminating polyps from healthy donors was 0.763 (95% CI: 0.699–0.828; *p* < 0.0001; Figure S2B). Furthermore, individuals with villous/tubulovillous (*p* < 0.0001) and adenoma/adenomatous (*p* < 0.01) polyps, categorised as having high cancer progression risk, displayed significantly elevated cSFRP5 levels compared to healthy controls (Figure S2C).

### 3.4 Circulating SFRP5 in other disease states

Comparing individuals with various medical conditions to healthy donors revealed elevated cSFRP5 levels among those with notable medical conditions, with a mean concentration of 21.23 ng/mL (95% CI: 17.84–24.62 ng/mL), significantly higher than healthy donors (*p* < 0.0001; Figure S3A). The AUROC curve for distinguishing between individuals with notable medical conditions and healthy donors yielded a value of 0.814 (95% CI: 0.746–0.882; *p* < 0.0001; Figure S3B). Moreover, cSFRP5 levels exhibited significant increases in individuals with other cancers (*p* < 0.0001), inflammation (*p* < 0.0001) and miscellaneous benign conditions (*p* < 0.01) in other organs compared to healthy controls (Figure S3C).

### 3.5 Elevated circulating SFRP5 levels were not exclusive to colorectal cancer

We employed a linear regression model to adjust for patient age, sex, plasma age and collection institution differences when comparing patient groups (Table S7). The adjusted cSFRP5 concentration was notably higher in patients with significant medical conditions (*p* < 0.0001), colorectal polyps (*p* = 0.0346), and CRC (*p* = 0.0063). Particularly, cSFRP5 levels were significantly elevated in patients with notable medical conditions compared to those with either colorectal polyps (*p* = 0.0059) or CRC (*p* = 0.0009). However, no significant difference was observed between the polyps and CRC groups. The AUROC curve for distinguishing between notable medical conditions and either CRC or polyps was 0.596 (95% CI: 0.518–0.674; *p* = 0.0127) and 0.613 (95% CI: 0.519–0.706; *p* = 0.0188), respectively (Figure S4). In summary, these findings suggest elevated cSFRP5 levels across various disease states, including colorectal polyps and CRC.

### 3.6 Circulating SFRP5 in colorectal cancer was associated with patient age, tumour stage and histological differentiation

In the patients with CRC, we investigated the correlations between the cSFRP5 concentration and different clinical‐pathological parameters (Table 2; Figure S5). We observed a significant increase in cSFRP5 concentration with patient age (*p* < 0.0001), TNM stage (*p* < 0.0001), advanced depth of invasion (*p* < 0.0001), in the absence of distant metastasis (*p* = 0.0049), and with poorly differentiated tumours (*p* = 0.0273). The cSFRP5 concentration was significantly higher in CRC patients with more invasive T3 and T4 primary tumours compared to the less invasive T1 and T2 (Figure S5A). Although there was an increase in cSFRP5 concentration in CRC with mismatch repair deficiency (dMMR; Figure S5I; *p* = 0.0655), it did not reach statistical significance. We did not detect variations in relation to sex, lymph node metastasis, vascular or perineural invasion, diabetes, or hypertension in the patients with CRC.

**TABLE 2:** cSFRP5 concentration in CRC with various prognostic clinical‐pathological parameters.

Clinical parameters	*n*	Mean cSFRP5 (95% CI)	*p*‐Value
Patient age^a^	449		**<0.0001**
Sex			
Female	172	17.65 (15.97–19.33)	0.3927
Male	277	17.69 (16.35–19.03)	
TNM stage			
I	147	14.18 (12.63–15.74)	**<0.0001**
II	103	23.94 (21.08–26.80)	
III	109	28.68 (16.96–20.41)	
IV	90	14.98 (13.26–16.70)	
Tumour invasion (T‐stage)			
T1	54	15.19 (12.11–18.27)	**<0.0001**
T2	103	13.72 (12.10–15.34)	
T3	175	21.15 (19.26–23.03)	
T4	58	19.15 (16.34–21.95)	
Lymph node metastasis (N‐stage)			
N0	260	18.06 (16.50–19.62)	0.2122
N1/N2	137	17.99 (16.49–19.49)	
NR	52		
Distant metastasis (M‐stage)			
M0	359	18.35 (17.13–19.57)	**0.0049**
M1	90	14.98 (13.26–16.70)	
Vascular or perineural invasion			
Negative	381	17.43 (16.28–18.57)	0.0980
Positive	68	19.07 (16.56–21.58)	
Histological differentiation			
Low (Grade 1–2)	374	17.06 (16.03–18.09)	**0.0273**
High (Grade 3–4)	75	20.74 (17.22–24.27)	
Tumour site			
Colon	311	18.07 (16.72–19.42)	0.7549
Rectum	138	16.78 (15.25–18.31)	
Mismatch repair status			
pMMR	220		0.0655
dMMR	24	18.21 (16.79–19.63)	
NR	205	25.20 (15.80–34.60)	
Diabetes			
Negative	130	17.66 (15.40–19.91)	0.2007
Positive	94	18.47 (16.43–20.50)	
NR	225		
Hypertension			
Negative	26	16.20 (13.93–18.46)	0.8196
Positive	198	18.23 (16.50–19.96)	
NR	225		

Abbreviations: 95% CI, 95% confidence interval; dMMR, mismatch repair deficiency; NR, not recorded; pMMR, mismatch repair proficient.

^a^ Analysed as a continuous variable.

Bold values indicates statisically significant *p*‐values.

### 3.7 Circulating SFRP5 correlates with overall survival in stage II‐III colorectal cancer

We examined the relationship between cSFRP5 and overall survival of all CRC patients using Cox proportional hazard models (Table S8). Follow‐up survival data were unavailable for 23 of 449 (5.1%) CRC patients (*n* = 6 TNM stage I, *n* = 4 stage II, *n* = 10 stage III and *n* = 3 stage IV); the proportion of patients lost to follow‐up did not significantly vary by TNM stage. The mean follow‐up period for the remaining patients was 4.3 years (range 1–249 months). Patients were stratified into low and high groups according to the median cSFRP5 concentration of 16.34 ng/mL observed among all CRC patients included in this study. When considering the entire CRC population encompassing stages I‐IV, no significant association emerged between cSFRP5 concentration and overall survival, as revealed by both univariate (*p* = 0.4866) and multivariable (*p* = 0.9721) models (Figure S6; Table S8).

**FIGURE 2:**
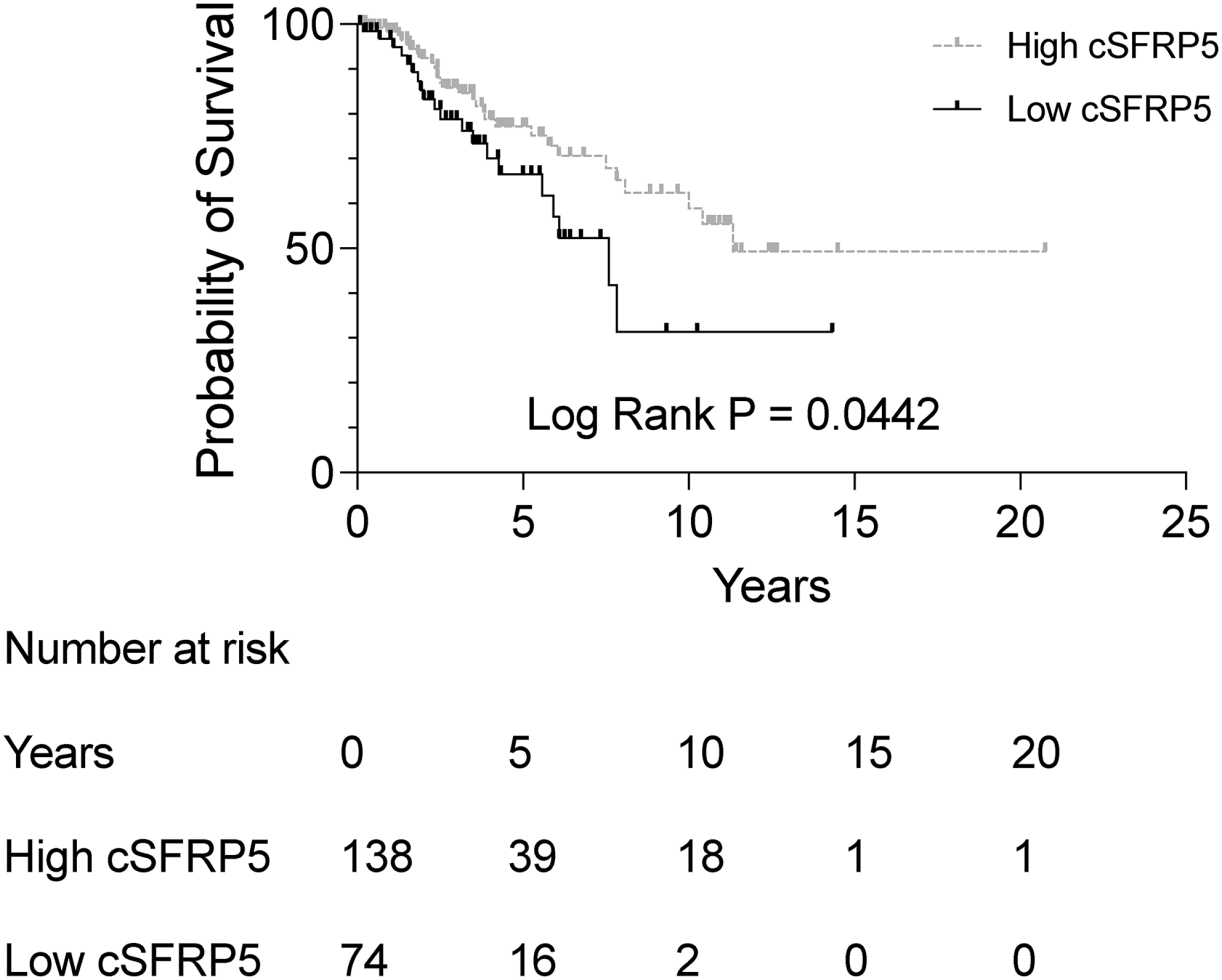
Kaplan–Meier survival curve for overall survival in patients with stage II‐III colorectal CRC (*n* = 212) dichotomized into low (≤16.34 ng/mL), or high (>16.34 ng/mL) cSFRP5 concentration.

Ultimately, we examined the relationship between cSFRP5 and overall survival among stage II‐III CRC patients, who are at an elevated risk of disease recurrence. High cSFRP5 levels were associated with extended overall survival (Figure 2; Table 3; HR 1.82; 95% CI: 1.02–3.26; *p* = 0.0442). This correlation persisted in multivariable analysis, accounting for confounding factors (HR 2.34; 95% CI: 1.12–4.88; *p* = 0.0229). These findings underscore the prognostic relevance of cSFRP5 in CRC, particularly in the context of stage II‐III disease.

**TABLE 3:** Univariate and multivariable analysis of overall survival in stage II‐III CRC.

Predictor	Univariate		Multivariable	
	Hazard ratio (95% CI)	*p*‐Value	Hazard ratio (95% CI)	*p*‐Value
cSFRP5				
High (>16.34 ng/mL)	1		1	
Low (≤16.34 ng/mL)	1.82 (1.02–3.26)	**0.044**	2.34 (1.12–4.88)	**0.023**
Patient age^a^	1.06 (1.03–1.09)	**<0.001**	1.07 (1.03–1.10)	**<0.001**
Sex				
Male	1		1	
Female	0.80 (0.44–1.45)	0.4609	0.81 (0.43–1.53)	0.521
TNM stage				
II	1		1	
III	1.41 (0.80–2.47)	0.2366	1.18 (0.61–2.27)	0.6151
Differentiation				
Low (Grade 1–2)	1		1	
High (Grade 3–4)	1.17 (0.63–2.16)	0.6203	1.03 (0.53–1.92)	0.992
Vascular or perineural invasion				
Negative	1		1	
Positive	1.67 (0.89–3.11)	0.1065	1.78 (0.89–3.55)	0.1003
Tumour site				
Colon	1		1	
Rectum	0.79 (0.43–1.43)	0.4344	0.95 (0.51–1.77)	0.8839

^a^ Analysed as a continuous variable.

Bold values indicates statisically significant *p*‐values.

## 4 DISCUSSION

This study investigated the levels of cSFRP5 across a diverse cohort, including healthy individuals, as well as those with CRC, colorectal polyps, and various other medical conditions. Elevated concentrations of cSFRP5 were identified in patients with CRC, polyps, and other significant illnesses, compared to the healthy controls. Notably, among CRC patients, adjusted cSFRP5 levels were highest in TNM stage II, followed by stage III and IV, and were lowest in stage I. Furthermore, cSFRP5 levels exhibited an increase with patient age and were higher in the poorly differentiated tumours. Moreover, high cSFRP5 levels were associated with longer overall survival in the stage II‐III CRC patients.

In our previous study, we highlighted the potential of cSFRP5 concentration as a diagnostic marker, effectively distinguishing CRC patients from those without CRC (AUROC 0.828, 95% CI: 0.761–0.898).^29^ In the current study we reported that the cSFRP5 level comparing patients with CRC to healthy donors had a AUROC of 0.762 (95% CI: 0.717–0.807; *p* < 0.0001). However, it is important to emphasise that we did not find a significant difference between patients with colorectal polyps and those with CRC. Noteworthy is the fact that most of these individuals with polyps have a high risk of developing into cancer. This suggests that cSFRP5 may hold value in identifying individuals with high‐risk polyps. However, our latest research revealed that individuals with other notable medical conditions also exhibited elevated cSFRP5 levels. These findings raise concerns about the exclusive use of cSFRP5 as a diagnostic biomarker for CRC population screening. However, it is important to note that the notable medical conditions group consisted of patients admitted to hospitals for treatment of various diseases, including cancer, inflammation, and benign states in organs other than the colorectum, and do not necessarily represent the general population.

Regarding CRC prognosis, our findings indicate that cSFRP5 levels are most elevated in patients with TNM stage II tumours, followed by stage III and IV, and are at their lowest in stage I, following adjustment for patient age, sex, plasma age and collection institution differences. This was similar to our previous findings in an independent CRC cohort, except that cSFRP5 levels were elevated in stages I, II and III compared to stage IV.^29^ It is important to note that the previous study was constrained by a smaller sample size (*n* = 100 CRC compared to *n* = 449 in this study). Additionally, we previously reported an association between elevated cSFRP5 and longer disease‐free survival (HR 2.385; 95% CI: 1.181–4.816; *p* = 0.015).^29^ Unfortunately, the current study lacked data on disease recurrence for a comprehensive analysis of disease‐free survival. However, the current observation that elevated cSFRP5 in CRC patients is linked to improved overall survival is consistent with the previous findings for disease‐free survival. Taken together, these findings suggest a favourable association between elevated cSFRP5 in CRC and survival outcomes, particularly in those with stage II‐III disease.

The explanation for the increased cSFRP5 observed in patients with CRC, polyps, and in the other disease states remains largely unclear. In CRC, it is well established that SFRP5 transcript expression decreases due to promoter DNA hypermethylation,^29^, ^30^ suggesting that the cancer cells are unlikely to be the origin of the heightened cSFRP5. However, to date there have been no studies that have reported SFRP5 protein levels in colorectal tissues. Research indicates that pancreatic cells express some of the highest levels of SFRP5.^29^, ^31^, ^32^ Elevated glucose and insulin levels have been shown to decrease SFRP5 expression in pancreatic cells,^33^ and insulin infusion leads to a decrease in serum cSFRP5.^34^ Conversely, free fatty acids counteract the insulin‐induced decrease in cSFRP5.^34^ Furthermore, serum cSFRP5 is positively correlated with HDL‐cholesterol and negatively correlated with triglycerides and adiponectin.^34^ Additionally, cSFRP5 is inversely associated with circulating levels of pro‐inflammatory cytokines, including IL‐6 and TNFα.^35^ These collective observations suggest that systemic disruptions in metabolism and inflammation drive changes in cSFRP5 levels, and potentially account for the elevated concentrations observed in the study.

It is well accepted that SFRP5 binds Wnt ligands to antagonise Wnt signalling pathways.^4^ The Wnt signalling pathway is intricate and contingent on context. This pathway is indispensable for normal cellular processes, including cell proliferation, differentiation, and tissue homeostasis. However, dysregulation of the Wnt pathway is a pivotal factor in the development and progression of CRC. Activation of the canonical Wnt/beta‐catenin signalling pathway is associated with poorer disease‐free, cancer‐specific and overall survival in CRC.^36^ Further, aberrantly activated Wnt/beta‐catenin signalling is associated with a T‐cell‐excluded phenotype, and inhibition of beta‐catenin shifts the colorectal tumour microenvironment into a T‐cell‐inflamed phenotype.^37^ Alternatively, the non‐canonical Wnt ligand, Wnt5a, is overexpressed in CRC and promotes cancer cell epithelial‐mesenchymal transition, facilitating metastasis.^38^, ^39^ The primary sources of Wnt5a in CRC are fibroblasts and macrophages. Hirashima et al. demonstrated that Wnt5a was expressed by cancer associated fibroblasts.^40^ The Wnt5a+ cancer associated fibroblasts were significantly associated with TNM stage, and recurrence. Subsequent in vitro analyses using human recombinant Wnt5a protein revealed that cancer cell proliferation and migration were significantly increased by stimulation with Wnt5a. Liu et al. identified that Wnt5a+ tumour‐associated macrophages were associated with disease progression and poor prognosis.^41^ Moreover, Wnt5a+ macrophages promoted CRC cell proliferation, invasion, and migration, and the knockdown of Wnt5a significantly impaired the pro‐tumour functions of tumour‐associated macrophages. We propose that the observed elevated levels of cSFRP5 contribute to inhibition of the Wnt‐signalling‐mediated disease progression. However, we acknowledge that further studies are required.

We noticed higher cSFRP5 levels in mismatch repair deficient CRC compared to proficient ones, but it didn't reach statistical significance. Unfortunately, only 244 out of 449 (54.3%) patients had their mismatch repair status reported, as it was not routinely tested at these institutions. Mismatch repair deficient CRC tends to have more mutations, generating neoantigens that attract immune cells to the tumour microenvironment. Meanwhile, SFRP5 by virtue of antagonising Wnt signalling pathways, has various effects on immune cells, influencing their behaviour and differentiation.^42^ It can shift macrophages toward an anti‐inflammatory type^12^ and affect the balance between effector and regulatory T cells, contributing to immune stability.^43^ Overall, SFRP5 appears to play a role in modulating immune cell differentiation, affecting the balance between pro‐inflammatory and anti‐inflammatory responses. More research is needed to determine the specific role of cSFRP5 in shaping the tumour immune cell microenvironment in CRC.

SFRP5 has garnered attention as a promising therapeutic target for various conditions, including metabolic diseases.^16^ Notably, SFRP5 administration reversed hyperglycaemia and hepatic steatosis in multiple mouse models of metabolic dysfunction.^12^ Lifestyle modifications leading to substantial weight loss in overweight individuals have been reported to increase cSFRP5 levels.^20^, ^21^, ^22^ Exercise training induced calorie expenditure in individuals with type 2 diabetes mellitus raised cSFRP5 levels.^44^ Additionally, clinically prescribed drugs such as the glucagon‐like peptide‐1 receptor agonist liraglutide have demonstrated the ability to elevate cSFRP5 levels.^45^ Considering the observed association of elevated cSFRP5 with improved disease‐free survival^29^ and overall survival, interventions that elevate cSFRP5 and target Wnt signalling pathways could emerge as attractive options for personalised medicine, particularly as an adjuvant therapy in individuals with stage II‐III CRC with a heightened risk of disease recurrence.

A significant limitation of this study is its retrospective design, which precludes adjustments to clinical practices. Additionally, the absence of complete data for potentially important confounders, such as hypertension, obesity, insulin resistance, and diabetes represent a noteworthy drawback. The unavailability of anthropometric measurements, such as body weight, height, and waist circumference, further constrains the comprehensiveness of our analysis. Moreover, the lack of information on therapeutic drug usage, such as chemotherapeutics and anti‐inflammatories, limits our ability to adequately assess these factors. Moving forward, the validation of our findings through prospective studies involving comprehensive CRC patient cohorts is crucial for advancing our understanding of the diagnostic and prognostic potential of cSFRP5.

Considering the observation that the adjusted cSFRP5 levels were highest in stage II, followed by stages III and IV, and lowest in stage I, and that high levels were associated with a favourable prognosis, it is important to acknowledge a limitation of our study. Specifically, we only measured cSFRP5 independently of other components of the highly complex and contextual Wnt‐signalling pathway. SFRP5 is one of five structurally similar family members. Based on their homology with the Wnt‐binding domain on the Frizzled receptors, SFRPs were initially characterised as antagonists that bind to various soluble Wnt ligands to prevent signal activation. However, subsequent studies have suggested that SFRPs may not only bind Wnt ligands to antagonise Wnt‐signalling but may also perform other functions, including antagonising one another's activity, potentiating Wnt‐signalling at lower concentrations, directly binding to Frizzled receptors, and interacting with receptors of other pathways and matrix molecules (reviewed in^4^). Future studies employing an integrative approach will be required to fully understand the observations of our study.

In conclusion, this study validated previous findings that cSFRP5 levels are elevated in CRC patients and revealed that the highest cSFRP5 concentrations were detected in TNM stage II, followed by stages III and IV, and the lowest in stage I, following adjustment for patient age, sex, plasma age and institution. Importantly, we report the novel finding that elevated cSFRP5 levels correlated with extended overall survival among stages II‐III CRC patients, emphasising its potential clinical significance as a prognostic biomarker. Our findings suggest that augmenting cSFRP5 levels could potentially enhance survival outcomes for stages II‐III CRC patients, who are most vulnerable to disease recurrence.

## AUTHOR CONTRIBUTIONS

**Runhao Li:** Conceptualization (lead); data curation (lead); formal analysis (lead); investigation (lead); methodology (lead); project administration (lead); software (lead); validation (lead); visualization (lead); writing – original draft (lead); writing – review and editing (lead). **Saifei Liu:** Conceptualization (equal); data curation (equal); investigation (equal); methodology (equal). **Kenny Yeo:** Data curation (equal); formal analysis (equal); software (equal). **Suzanne Edwards:** Formal analysis (equal); software (equal). **Man Ying Li:** Data curation (equal); methodology (equal); resources (equal). **Ryan Santos:** Investigation (equal); methodology (equal). **Sima Kianpour Rad:** Data curation (equal); investigation (equal); methodology (equal). **Fangmeinuo Wu:** Investigation (equal); methodology (equal). **Guy Maddern:** Funding acquisition (equal); project administration (equal); resources (equal); supervision (equal); writing – review and editing (equal). **Joanne Young:** Project administration (equal); supervision (equal); writing – review and editing (equal). **Yoko Tomita:** Project administration (equal); supervision (equal); writing – review and editing (equal). **Amanda Townsend:** Project administration (equal); supervision (equal); writing – review and editing (equal). **Kevin Fenix:** Conceptualization (equal); funding acquisition (equal); project administration (equal); supervision (equal); writing – review and editing (equal). **Ehud Hauben:** Conceptualization (equal); funding acquisition (equal); project administration (equal); supervision (equal); writing – review and editing (equal). **Timothy Price:** Conceptualization (equal); funding acquisition (lead); project administration (equal); supervision (equal); writing – review and editing (equal). **Eric Smith:** Conceptualization (lead); funding acquisition (lead); investigation (lead); methodology (lead); project administration (lead); supervision (lead); writing – original draft (lead); writing – review and editing (lead).

## FUNDING INFORMATION

This work was supported by AusHealth (Grant number RES‐SFRP‐01). Author Runhao Li has received an International PhD scholarship from The University of Adelaide.

## CONFLICT OF INTEREST STATEMENT

The authors have no relevant financial or non‐financial interests to disclose.

## ETHICS SATATEMENT

This study was performed in line with the principles of the Declaration of Helsinki, and in accordance with the Australian Code for the Responsible Conduct of Research and the National Statement on Ethical Conduct in Human Research. Approval was granted by the Central Adelaide Local Health Network Human Research Ethics Committee (Date 21‐Sep‐2021/No. HREC/14/TQEHLMH/164).

## CONSENT

Informed consent was obtained from all individual participants included in the study.

## Supporting information


Data S1:

## Data Availability

The data that support the findings of this study are available from the corresponding author upon reasonable request.

## References

[1] Sung H , Ferlay J , Siegel RL , et al. Global cancer statistics 2020: GLOBOCAN estimates of incidence and mortality worldwide for 36 cancers in 185 countries. CA Cancer J Clin. 2021;71(3):209‐249. doi:10.3322/caac.21660 33538338

[2] Kastrinos F , Kupfer SS , Gupta S . Colorectal cancer risk assessment and precision approaches to screening: brave new world or worlds apart? Gastroenterology. 2023;164(5):812‐827. doi:10.1053/j.gastro.2023.02.021 36841490 PMC10370261

[3] Mikaeel RR , Symonds EL , Kimber J , et al. Young‐onset colorectal cancer is associated with a personal history of type 2 diabetes. Asia Pac J Clin Oncol. 2021;17(1):131‐138. doi:10.1111/ajco.13428 32885561

[4] Bovolenta P , Esteve P , Ruiz JM , Cisneros E , Lopez‐Rios J . Beyond Wnt inhibition: new functions of secreted frizzled‐related proteins in development and disease. J Cell Sci. 2008;121(Pt 6):737‐746. doi:10.1242/jcs.026096 18322270

[5] Komiya Y , Habas R . Wnt signal transduction pathways. Organogenesis. 2008;4(2):68‐75. doi:10.4161/org.4.2.5851 19279717 PMC2634250

[6] Klaus A , Birchmeier W . Wnt signalling and its impact on development and cancer. Nat Rev Cancer. 2008;8(5):387‐398. doi:10.1038/nrc2389 18432252

[7] Anastas JN , Moon RT . WNT signalling pathways as therapeutic targets in cancer. Nat Rev Cancer. 2013;13(1):11‐26. doi:10.1038/nrc3419 23258168

[8] Jones SE , Jomary C . Secreted frizzled‐related proteins: searching for relationships and patterns. BioEssays. 2002;24(9):811‐820. doi:10.1002/bies.10136 12210517

[9] Bhat RA , Stauffer B , Komm BS , Bodine PV . Structure‐function analysis of secreted frizzled‐related protein‐1 for its Wnt antagonist function. J Cell Biochem. 2007;102(6):1519‐1528. doi:10.1002/jcb.21372 17471511

[10] Bányai L , Patthy L . The NTR module: domains of netrins, secreted frizzled related proteins, and type I procollagen C‐proteinase enhancer protein are homologous with tissue inhibitors of metalloproteases. Protein Sci. 1999;8(8):1636‐1642. doi:10.1110/ps.8.8.1636 10452607 PMC2144412

[11] Schulte G . International Union of Basic and Clinical Pharmacology. LXXX. The class frizzled receptors. Pharmacol Rev. 2010;62(4):632‐667. doi:10.1124/pr.110.002931 21079039

[12] Ouchi N , Higuchi A , Ohashi K , et al. Sfrp5 is an anti‐inflammatory adipokine that modulates metabolic dysfunction in obesity. Science. 2010;329(5990):454‐457. doi:10.1126/science.1188280 20558665 PMC3132938

[13] Li Y , Rankin SA , Sinner D , Kenny AP , Krieg PA , Zorn AM . Sfrp5 coordinates foregut specification and morphogenesis by antagonizing both canonical and noncanonical Wnt11 signaling. Genes Dev. 2008;22(21):3050‐3063. doi:10.1101/gad.1687308 18981481 PMC2577796

[14] Chatani N , Kamada Y , Kizu T , et al. Secreted frizzled‐related protein 5 (Sfrp5) decreases hepatic stellate cell activation and liver fibrosis. Liver Int. 2015;35(8):2017‐2026. doi:10.1111/liv.12757 25488180

[15] Catalán V , Gómez‐Ambrosi J , Rodríguez A , et al. Activation of noncanonical Wnt signaling through WNT5A in visceral adipose tissue of obese subjects is related to inflammation. J Clin Endocrinol Metab. 2014;99(8):E1407‐E1417. doi:10.1210/jc.2014-1191 24840810

[16] Song Y , Ma Y , Zhang K , et al. Secreted frizzled‐related protein 5: a promising therapeutic target for metabolic diseases via regulation of Wnt signaling. Biochem Biophys Res Commun. 2023;677:70‐76. doi:10.1016/j.bbrc.2023.08.008 37549604

[17] Wang D , Zhang Y , Shen C . Research update on the association between SFRP5, an anti‐inflammatory adipokine, with obesity, type 2 diabetes mellitus and coronary heart disease. J Cell Mol Med. 2020;24(5):2730‐2735. doi:10.1111/jcmm.15023 32004418 PMC7077606

[18] Tong S , Ji Q , Du Y , Zhu X , Zhu C , Zhou Y . Sfrp5/Wnt pathway: a protective regulatory system in atherosclerotic cardiovascular disease. J Interf Cytokine Res. 2019;39(8):472‐482. doi:10.1089/jir.2018.0154 PMC6660834 31199714

[19] Nakamura K , Fuster JJ , Walsh K . Adipokines: a link between obesity and cardiovascular disease. J Cardiol. 2014;63(4):250‐259. doi:10.1016/j.jjcc.2013.11.006 24355497 PMC3989503

[20] Tan X , Wang X , Chu H , Liu H , Yi X , Xiao Y . SFRP5 correlates with obesity and metabolic syndrome and increases after weight loss in children. Clin Endocrinol. 2014;81(3):363‐369. doi:10.1111/cen.12361 24330025

[21] Yin C , Chu H , Li H , Xiao Y . Plasma Sfrp5 and adiponectin levels in relation to blood pressure among obese children. J Hum Hypertens. 2017;31(4):284‐291. doi:10.1038/jhh.2016.76 27882931

[22] Schulte DM , Müller N , Neumann K , et al. Pro‐inflammatory wnt5a and anti‐inflammatory sFRP5 are differentially regulated by nutritional factors in obese human subjects. PLoS One. 2012;7(2):e32437. doi:10.1371/journal.pone.0032437 22384249 PMC3285685

[23] Bhome R , Peppa N , Karar S , McDonnell D , Mirnezami A , Hamady Z . Metabolic syndrome is a predictor of all site and liver‐specific recurrence following primary resection of colorectal cancer: prospective cohort study of 1006 patients. Eur J Surg Oncol. 2021;47(7):1623‐1628. doi:10.1016/j.ejso.2020.12.016 33483238 PMC7611058

[24] Cho YK , Kang YM , Lee SE , et al. Effect of SFRP5 (secreted frizzled‐related protein 5) on the WNT5A (wingless‐type family member 5A)‐induced endothelial dysfunction and its relevance with arterial stiffness in human subjects. Arterioscler Thromb Vasc Biol. 2018;38(6):1358‐1367. doi:10.1161/atvbaha.117.310649 29674475

[25] Wang B , Pan Y , Yang G , et al. Sfrp5/Wnt5a and leptin/adiponectin levels in the serum and the periarterial adipose tissue of patients with peripheral arterial occlusive disease. Clin Biochem. 2021;87:46‐51. doi:10.1016/j.clinbiochem.2020.11.002 33188773

[26] Miyoshi T , Doi M , Usui S , et al. Low serum level of secreted frizzled‐related protein 5, an anti‐inflammatory adipokine, is associated with coronary artery disease. Atherosclerosis. 2014;233(2):454‐459. doi:10.1016/j.atherosclerosis.2014.01.019 24530778

[27] Tong S , Du Y , Ji Q , et al. Expression of Sfrp5/Wnt5a in human epicardial adipose tissue and their relationship with coronary artery disease. Life Sci. 2020;245:117338. doi:10.1016/j.lfs.2020.117338 31981630

[28] Li B , Yao Q , Guo S , et al. Type 2 diabetes with hypertensive patients results in changes to features of adipocytokines: leptin, irisin, LGR4, and Sfrp5. Clin Exp Hypertens. 2019;41(7):645‐650. doi:10.1080/10641963.2018.1529779 30307757

[29] Kirana C , Smith E , Ngo DT , et al. High preoperative levels of circulating SFRP5 predict better prognosis in colorectal cancer patients. Future Oncol. 2020;16(31):2499‐2509. doi:10.2217/fon-2020-0356 33048585

[30] Qi J , Zhu YQ , Luo J , Tao WH . Hypermethylation and expression regulation of secreted frizzled‐related protein genes in colorectal tumor. World J Gastroenterol. 2006;12(44):7113‐7117. doi:10.3748/wjg.v12.i44.7113 17131472 PMC4087771

[31] Tabula Muris Consortium , Overall coordination , Logistical coordination , et al. Single‐cell transcriptomics of 20 mouse organs creates a Tabula Muris. Nature. 2018;562(7727):367‐372. doi:10.1038/s41586-018-0590-4 30283141 PMC6642641

[32] Jones RC , Karkanias J , Krasnow MA , et al. The tabula sapiens: a multiple‐organ, single‐cell transcriptomic atlas of humans. Science. 2022;376(6594):eabl4896. doi:10.1126/science.abl4896 35549404 PMC9812260

[33] Guan B , Li W , Li F , et al. Sfrp5 mediates glucose‐induced proliferation in rat pancreatic β‐cells. J Endocrinol. 2016;229(2):73‐83. doi:10.1530/joe-15-0535 26869333

[34] Rydzewska M , Nikołajuk A , Matulewicz N , Stefanowicz M , Karczewska‐Kupczewska M . Serum secreted frizzled‐related protein 5 in relation to insulin sensitivity and its regulation by insulin and free fatty acids. Endocrine. 2021;74(2):300‐307. doi:10.1007/s12020-021-02793-z 34184187 PMC8497315

[35] Zhang Y , Ran Y , Kong L , et al. Decreased SFRP5 correlated with excessive metabolic inflammation in polycystic ovary syndrome could be reversed by metformin: implication of its role in dysregulated metabolism. J Ovarian Res. 2021;14(1):97. doi:10.1186/s13048-021-00847-4 34284806 PMC8293500

[36] Matly A , Quinn JA , McMillan DC , Park JH , Edwards J . The relationship between β‐catenin and patient survival in colorectal cancer systematic review and meta‐analysis. Crit Rev Oncol Hematol. 2021;163:103337. doi:10.1016/j.critrevonc.2021.103337 33992802

[37] Wang C , Yan J , Yin P , et al. β‐Catenin inhibition shapes tumor immunity and synergizes with immunotherapy in colorectal cancer. Onco Targets Ther. 2020;9(1):1809947. doi:10.1080/2162402x.2020.1809947 PMC7470182 32939327

[38] Gujral TS , Chan M , Peshkin L , Sorger PK , Kirschner MW , MacBeath G . A noncanonical Frizzled2 pathway regulates epithelial‐mesenchymal transition and metastasis. Cell. 2014;159(4):844‐856. doi:10.1016/j.cell.2014.10.032 25417160 PMC4243058

[39] Fuertes G , Del Valle‐Pérez B , Pastor J , et al. Noncanonical Wnt signaling promotes colon tumor growth, chemoresistance and tumor fibroblast activation. EMBO Rep. 2023;24(4):e54895. doi:10.15252/embr.202254895 36704936 PMC10074097

[40] Hirashima T , Karasawa H , Aizawa T , et al. Wnt5a in cancer‐associated fibroblasts promotes colorectal cancer progression. Biochem Biophys Res Commun. 2021;568:37‐42. doi:10.1016/j.bbrc.2021.06.062 34175688

[41] Liu Q , Yang C , Wang S , et al. Wnt5a‐induced M2 polarization of tumor‐associated macrophages via IL‐10 promotes colorectal cancer progression. Cell Commun Signal. 2020;18(1):51. doi:10.1186/s12964-020-00557-2 32228612 PMC7106599

[42] Haseeb M , Pirzada RH , Ain QU , Choi S . Wnt signaling in the regulation of immune cell and cancer therapeutics. Cells. 2019;8(11):1380. doi:10.3390/cells8111380 31684152 PMC6912555

[43] Pundkar C , Antony F , Kang X , et al. Targeting Wnt/β‐catenin signaling using XAV939 nanoparticles in tumor microenvironment‐conditioned macrophages promote immunogenicity. Heliyon. 2023;9(6):e16688. doi:10.1016/j.heliyon.2023.e16688 37313143 PMC10258387

[44] Zadeh MAM , Afrasyabi S , Mohamadi ZA . The effects of exercise training induced calories expenditure on type 2 diabetes related cardio metabolic physiological parameters and adipocytokines. J Diabetes Metab Disord. 2022;21(2):1219‐1231. doi:10.1007/s40200-021-00808-0 36404859 PMC9672291

[45] Hu W , Li L , Yang M , et al. Circulating Sfrp5 is a signature of obesity‐related metabolic disorders and is regulated by glucose and liraglutide in humans. J Clin Endocrinol Metab. 2013;98(1):290‐298. doi:10.1210/jc.2012-2466 23185036

