# Supplementary material for: Diagnostic and prognostic significance of circulating secreted frizzled‐related protein 5 in colorectal cancer
Source: Cancer Med. 2024 Jun 14;13(11):e7352. doi: 10.1002/cam4.7352 (PMC11176579; doi:10.1002/cam4.7352)
Supplement: Supplementary file 1 — Data S1: [file CAM4-13-e7352-s001.docx]

**SUPPLEMENTARY INFORMATION**

**Supplementary fig. 1:** Patient age and gender by donor groups, and cSFRP5 concentration by collection healthcare institution. (**A**) Comparison of patient age in healthy donors without documented pathology (H) (n=133), patients with other notable medical conditions (D) (n=64), colorectal polyps (P) (n=85), or colorectal cancer (CRC) patients (n=449): stage I (n=147), stage II (n=103), stage III (n=109), and stage IV (n=90). (**B**) Comparison of gender ratio between different groups. **(C-F)** Concentration of cSFRP5 by collection healthcare institution in the different donor groups. Data as mean ± SD. Kruskal-Wallis with Dunn’s multiple comparisons were used, ****p<0.0001, ***p<0.001, **p<0.01, *p<0.05.

**Supplementary fig. 2**: Concentration of cSFRP5 in healthy donors (H) and patients with colorectal polyps (P) (**A**) Comparison of cSFRP5 concentration in H (n=133) and P (n=85). (**B**) AUROC curve for cSRFP5 concentration comparing H to P. (**C**) Concentration of cSFRP5 in healthy donors (H) and patients with villous/tubulovillous (n=48), adenoma/adenomatous (n=31), sessile serrated (n=2), and other (n=4) types of colorectal polyps. The data are the mean ± SD**.** Kruskal-Wallis with Dunn’s multiple comparisons were used, ****p<0.0001, **p<0.01.

**Supplementary fig. 3**: Concentration of cSFRP5 in healthy donors (H) and other notable medical conditions (D). (**A**) Comparison of cSFRP5 concentration in H (n=133) and D (n=64). (**B**) AUROC curve for cSRFP5 concentration comparing H to D. (**C**) Concentration of cSFRP5 in healthy donors (H) (n=133) and patients with different types of notable medical conditions or significant disease by benign diseases (n=11), chronic inflammation (n=49), and cancer (n=4). The data are the mean ± SD**.** Kruskal-Wallis with Dunn’s multiple comparisons were used, ****p<0.0001, ***p<0.001, **p<0.01, *p<0.05.

**Supplementary fig. 4:** cSFRP5 AUROC curves for (**A**) colorectal cancer polyps (P) (n=85) versus colorectal cancer (CRC) (n=449), (**B**) other notable medical conditions (D) (n=64) versus CRC (n=449), and (**C**) P (n=85) versus D (n=64).

**Supplementary fig. 5:** Concentration of cSFRP5 by various prognostic clinical-pathological parameters. (**A**) primary tumour invasion (T) stage. T1: tumour invades submucosa (n=54), T2: tumour invades muscularis propria (n=103), T3: tumour invades through the muscularis propria into peri-colorectal tissues (n=175), and T4: tumour directly invades or is adherent to other organs or structures (n=58). (**B-K**) cSRFP5 concentration comparison of sex, lymph node metastasis, distant metastasis, vascular or perineural invasion (VPNI), differentiation, tumour site, mismatch repair status, diabetes, and hypertension respectively. The data are the mean ± SD. A linear regression model was used for multiple comparisons, ****p<0.0001, ***p<0.001, **p<0.01, *p<0.05.

**Supplementary fig. 6:** The Kaplan-Meier survival curves for overall survival in patients with colorectal cancer based on low and high cSFRP5 expression levels. (**A**) All patients (n=449), (**B**) Stage I (n=147), (**C**) Stage II (n=103), (**D**) Stage III (n=109), (**E**) Stage IV (n=90). Patients are dichotomized into low (≤16.34 ng/mL), or high (>16.34 ng/mL) cSFRP5 concentration, and survival curve were compared using Kaplan-Meier plots.

**Supplementary Table 1** Summary of all samples from different institutions

| **Institution** | Healthy (H) | Notable medical conditions (D) | Colorectal polyps (P) | Colorectal Cancer (CRC) | **Total (n)** |
| --- | --- | --- | --- | --- | --- |
| Austin Hospital (AH) | 21 | 26 | 8 | 85 | 140 |
| Barwon Health (BH) | - | - | 3 | 1 | 4 |
| Eastern Health (EH) | 6 | 11 | 20 | 184 | 221 |
| Monash Health (MH) | - | - | 4 | 5 | 9 |
| North Health (NH) | 2 | 6 | 5 | 3 | 16 |
| Peter MacCallum Cancer Centre (PM) | 104 | 21 | 15 | 86 | 226 |
| Royal Melbourne Hospital (RMH) | - | - | 13 | 20 | 33 |
| Western Health (WH) | - | - | 17 | 65 | 82 |
| **Total** | **133** | **64** | **85** | **449** | **731** |

**Supplementary Table 2** Summary of healthy donors without significant documented pathology

| **Group** | **n** | **Female (%)** | **Median age, years (range)** |
| --- | --- | --- | --- |
| Healthy | 75 | 28 (37.3%) | 41 (16-80) |
| Prophylactic mastectomy | 37 | 37 (100%) | 41 (22-60) |
| Breast reduction | 19 | 19 (100%) | 43 (18-64) |
| Uterus endocervical polyp | 1 | 1 (100%) | 63 |
| Lipoma | 1 | 1 (100%) | 68 |
| **Total** | **133** | **86 (50.0%)** | **42 (16-80)** |

**Supplementary Table 3** Summary of patients with colorectal polyps

| **Polyp type** | **n** |
| --- | --- |
| Villous/tubulovillous adenoma | 48 |
| Adenoma/adenomatous | 31 |
| Sessile serrated | 2 |
| Hyperplastic | 2 |
| Inflammatory | 1 |
| Peutz Jegher syndrome | 1 |
| **Total** | **85** |

**Supplementary Table 4** Summary of patients with notable medical conditions

| **Classification** | **Notable medical conditions** | **n** | **Total** |
| --- | --- | --- | --- |
| Cancer | Lobectomy for lung cancer | 1 | 4 |
|  | Osteosarcoma; abscess | 1 |  |
|  | Renal oncocytoma | 1 |  |
|  | Transitional cell carcinoma | 1 |  |
| Chronic inflammation | Active chronic colitis/proctitis | 1 | 49 |
|  | Bladder wall lesions | 1 |  |
|  | Bronchiolitis & pneumonia | 11 |  |
|  | Cholangitis and pancreatitis | 1 |  |
|  | Cholecystitis and cholelithiasis | 8 |  |
|  | Cirrhotic appearance | 1 |  |
|  | Crohn's disease | 2 |  |
|  | Diverticulosis | 4 |  |
|  | Duodenal oedema and mild inflammation | 1 |  |
|  | Hashimoto's (autoimmune) thyroiditis | 3 |  |
|  | Interstitial cystitis | 1 |  |
|  | Lung cavitating lesion | 1 |  |
|  | Necrotizing granulomatous lymphadenitis | 1 |  |
|  | Non-necrotising granulomatous inflammation | 1 |  |
|  | Peritoneal abscess | 1 |  |
|  | Pleural bloodstained fluid | 1 |  |
|  | Prostatitis | 5 |  |
|  | Reactive lymphoid hyperplasia | 1 |  |
|  | Resection of ileoanal pouch | 1 |  |
|  | Right hemicolectomy: ulceration | 1 |  |
|  | Sinus histiocytosis | 1 |  |
|  | Splenectomy, autoimmune hemolytic anaemia | 1 |  |
| Benign disease | Meckel's diverticulum | 1 | 11 |
|  | Peri-rectal bleeding | 1 |  |
|  | Haematuria, likely benign prostatic hyperplasia | 1 |  |
|  | Benign multinodular goitre | 2 |  |
|  | Multiple simple bilateral renal cysts | 1 |  |
|  | Nodular and prostatic hyperplasia | 2 |  |
|  | Anthracosis | 1 |  |
|  | Haematoma | 1 |  |
|  | Hydronephrosis | 1 |  |

**Supplementary Table 5** Correlation analysis between concentration of cSFRP5 and plasma age (years) in different groups.

|  | H | D | P | CRC stage I | CRC stage II | CRC stage III | CRC stage IV |
| --- | --- | --- | --- | --- | --- | --- | --- |
| Spearman r | -0.0795 | 0.0732 | 0.0739 | 0.0180 | -0.0072 | -0.0698 | 0.1306 |
| 95% CI | -0.2464 to 0.0919 | -0.1758 to 0.3134 | -0.1415 to 0.2825 | -0.1443 to 0.1793 | -0.2004 to 0.1866 | -0.2546 to 0.1198 | -0.0785 to 0.3288 |
| r-squared | 0.0063 | 0.0054 | 0.0055 | 0.0003 | 0.0001 | 0.0049 | 0.0171 |
| P value (two-tailed) | 0.3630 | 0.5653 | 0.5017 | 0.8289 | 0.9426 | 0.4705 | 0.2197 |
| Number | 133 | 64 | 85 | 147 | 103 | 109 | 90 |

**Supplementary Table 6** Linear regression model of cSFRP5 (continuous) versus TNM stage

| **Predictor** | **Comparison** | **Unadjusted** | | **Adjusted by patient age, sex, institution, and plasma age** | |
| --- | --- | --- | --- | --- | --- |
|  |  | **Mean difference (95% CI)** | **P value** | **Mean difference (95% CI)** | **P value** |
| TNM stage | I vs II | -9.76 (-12.42, -7.10) | **<0.0001** | -10.30 (-12.99, -7.62) | **<0.0001** |
|  | I vs III | -4.50 (-7.12, -1.89) | **0.0007** | -5.02 (-7.70, -2.33) | **0.0002** |
|  | I vs IV | -0.80 (-3.57, 1.97) | 0.5725 | -3.02 (-5.93, -0.12) | **0.0413** |
|  | II vs III | 5.26 (2.41, 8.10) | **0.0003** | 5.29 (2.46, 8.11) | **0.0002** |
|  | II vs IV | 8.96 (5.98, 11.95) | **<0.0001** | 7.28 (4.16, 10.40) | **<0.0001** |
|  | III vs IV | 5.10 (1.69, 8.55) | **0.0054** | 1.99 (-1.06, 5.05) | 0.2015 |
|  |  |  |  |  | **Global P value** |
| Patient age | Per 10-year increase |  |  | 1.48 (0.68, 2.27) | **0.0003** |
| Sex | Female vs Male |  |  | -0.002 (-1.99, 1.98) | 0.9977 |
| Plasma age | Per 1-year increase |  |  | -0.01 (-0.26, 0.24) | 0.9408 |
| Institution |  |  |  |  | 0.2469 |

**Supplementary Table 7** Linear regression model of cSFRP5 (continuous) versus patient groups

| **Predictor** | **Comparison** | **Unadjusted** | | **Adjusted by patient age, sex, institution, and plasma age** | |
| --- | --- | --- | --- | --- | --- |
|  |  | **Mean difference (95% CI)** | **P value** | **Mean difference (95% CI)** | **P value** |
| Patient group | H vs D | -11.97 (-15.17, -8.76) | **<0.0001** | -8.72 (-12.22, -5.23) | **<0.0001** |
|  | H vs P | -8.08 (-11.00, -5.15) | **<0.0001** | -3.67 (-7.07, -0.27) | **0.0346** |
|  | H vs CRC | -8.41 (-10.49, -6.33) | **<0.0001** | -3.71 (-6.36, -1.05) | **0.0063** |
|  | D vs P | 3.89 (0.40, 7.37) | **0.0288** | 5.06 (1.46, 8.66) | **0.0059** |
|  | D vs CRC | 3.56 (0.74, 6.37) | **0.0132** | 5.02 (2.07, 7.97) | **0.0009** |
|  | P vs CRC | -0.33 (-2.82, 2.16) | 0.7949 | -0.04 (-2.60, 2.52) | 0.9759 |
|  |  |  |  |  | **Global P value** |
| Patient age | Per 10-year increase |  |  | 1.19 (0.61, 1.78) | **<0.0001** |
| Sex | Female vs Male |  |  | -0.98 (-2.59, 0.62) | 0.2294 |
| Plasma age | Per 1-year increase |  |  | 0.01 (-0.17, 0.20) | 0.9108 |
| Institution |  |  |  |  | 0.1716 |

**Supplementary Table 8** Univariate and multivariable analysis of overall survival for all CRC patients

| **Predictor** | **Univariate** | | **Multivariable** | |
| --- | --- | --- | --- | --- |
|  | **Hazard ratio (95% CI)** | **P value** | **Hazard ratio (95% CI)** | **P value** |
| **cSFRP5*** | 1.01 (0.99-1.02) | 0.4866 | 0.99 (0.98-1.02) | 0.9721 |
| **Patient age*** | 1.03 (1.02-1.05) | **<0.001** | 1.06 (1.04-1.08) | **<0.001** |
| **Sex**  Male  Female | 1  0.86 (0.61-1.22) | 0.4066 | 1  1.06 (0.74-1.53) | 0.7331 |
| **TNM stage**  I  II  III  IV | 1  1.33 (0.78-2.27)  1.91 (1.13-3.21)  5.56 (3.55-8.72) | 0.2894  **0.0151**  **<0.001** | 1  1.24 (0.70-2.19)  1.69 (0.94-3.05)  8.58 (5.16-14.25) | 0.4526  0.0943  **<0.001** |
| **Differentiation**  Low (Grade 1-2)  High (Grade 3-4) | 1  1.23 (0.81-1.86) | 0.3286 | 1  1.15 (0.74-1.79) | 0.5442 |
| **Vascular or perineural invasion**  Negative  Positive | 1  2.27 (1.51-3.42) | **<0.001** | 1  1.59 (1.02-2.49) | **0.0402** |
| **Tumour site**  Colon  Rectum | 1  0.72 (0.49-1.07) | 0.1049 | 1  1.25 (0.81-1.91) | 0.3084 |

**Analysed as a continuous variable.*
